# Improving the evidence base of Markov models used to estimate the costs of scaling up antiretroviral programmes in resource-limited settings

**DOI:** 10.1186/1472-6963-10-S1-S3

**Published:** 2010-07-02

**Authors:** Rory Leisegang, Gary Maartens, Michael Hislop, Leon Regensberg, Susan Cleary

**Affiliations:** 1 Division of Clinical of Pharmacology, Department of Medicine, University of Cape Town, Cape Town, South Africa; 2 Aid for AIDS, Division of Medscheme Holdings Pty Ltd, Cape Town, South Africa; 3 Health Economics Unit, School of Public Health and Family Medicine, University of Cape Town, Cape Town, South Africa

## Abstract

**Background:**

Despite concerns about affordability and sustainability, many models of the lifetime costs of antiretroviral therapy (ART) used in resource limited settings are based on data from small research cohorts, together with pragmatic assumptions about life-expectancy. This paper revisits these modelling assumptions in order to provide input to future attempts to model the lifetime costs and the costs of scaling up ART.

**Methods:**

We analysed the determinants of costs and outcomes in patients receiving ART in line with standard World Health Organization (WHO) guidelines for resource poor settings in a private sector managed ART programme in South Africa. The cohort included over 5,000 patients with up to 4 years (median 19 months) on ART. Generalized linear and Cox proportional hazards regression models were used to establish cost and outcome determinants respectively.

**Results:**

The key variables associated with changes in mean monthly costs were: being on the second line regimen; receiving ART from 4 months prior to 4 months post treatment initiation; having a recent or current CD4 count <50 cells/µL or 50-199 cells/µl; having mean ART adherence <75% as determined by monthly pharmacy refill data; and having a current or recent viral load >100,000 copies/mL. In terms of the likelihood of dying, the key variables were: baseline CD4 count<50 cells/µl (particularly during the first 4 months on treatment); current CD4 count <50 cells/µl and 50-199 cells/µl (particularly during later periods on treatment); and being on the second line regimen. Being poorly adherent and having an unsuppressed viral load was also associated with a higher likelihood of dying.

**Conclusions:**

While there are many unknowns associated with modelling the resources needed to scale-up ART, our analysis has suggested a number of key variables which can be used to improve the state of the art of modelling ART. While the magnitude of the effects associated with these variables would be likely to differ in other settings, the variables influencing costs and survival are likely to be generalizable. This is of direct relevance to those concerned about assessing the long-term costs and sustainability of expanded access to ART.

## Introduction

With access to antiretroviral therapy (ART) now rapidly expanding in low and middle-income countries, attention is increasingly turning to the affordability and sustainability of these programmes [1]. Given the potential effectiveness of treatment coupled with the scale of the response needed, it is important that planning takes a long term perspective. While many studies have focussed on the effectiveness of ART in resource-limited settings, cost studies are limited, especially those documenting costs in routine and established programmes and over longer periods of time. In recent years, the management of ART programmes in low and middle income countries has increasingly conformed to the World Health Organization (WHO) guidelines for resource-limited settings [2]. These include guidelines for when to start ART based on the patient’s CD4 count or WHO stage, guidelines for monitoring ART as well as guidelines regarding which antiretrovirals (ARVs) should be administered within distinct first and second line regimens. These guidelines therefore provide a good framework for understanding disease progression and the costs of patients in ART programmes.

Because ART has only recently been available in resource-limited settings, lifetime costs – a key input into the costs of scaling up - are calculated through extrapolating primary data, with the Markov model being the most common framework used for this extrapolation. Many models include the baseline and current CD4+ cell count (i.e. the most recent test value), viral load and WHO staging, but other potential determinants of costs such as adherence have been excluded. This raises questions of the accuracy of the resulting estimates which could have implications for attempts to plan for expanded access to ART.

A Markov model consists of a number of mutually exclusive and collectively exhaustive Markov states, with at least one of these being an “absorbing state” (e.g. death). Patients remain in each state for an equal increment of time, called a Markov cycle, before being allowed the option of moving to a different state (or staying in the current state) as determined by one or more transition probabilities. In addition to time (or survival) increments, health care costs are attached to each state. Over a large number of cycles, lifetime costs and life expectancy is estimated [3,4].

To establish appropriate Markov states it is thus necessary to estimate which variables have a sizeable impact on the costs associated with being in a state together with the transition probabilities determining movements between states. While many types of transition probabilities are possible, the most important is the probability of dying as this determines overall life expectancy. Because the majority of the costs of ART are associated with ARV drugs, accurate calculation of life expectancy is crucial for the estimation of lifetime costs which in turn is a key input into calculations of the costs of scaling up [5].

This paper seeks to identify the variables that have an impact on direct health care costs and the likelihood of dying with a view to informing the development of Markov models for estimating lifetime costs and the costs of scaling up ART in resource-limited settings. We initially review the ART Markov model and cost determinant literature to establish the variables and variable categories that have been used to date. Thereafter, we assess the importance of these through the analysis of a large cohort from a private health care disease management programme. While this analysis would ideally be conducted using data from individuals receiving ART in a range of routine models of care, including those found in the public health care sector, these routine data are not available. We have attempted to improve the generalisability of our findings by restricting our analysis to those patients in the private disease management programme that receive ART in line with the WHO guidelines for resource-limited settings [2].

## Methods

### Literature review

Our literature review included all cost, cost-effectiveness and cost-utility analyses of HIV-treatment including ART in resource-poor settings. While most economic analyses of ART focus on the annual per patient cost or the cost per specified outcome measure (e.g. per patient virally suppressed), we restricted our review to studies that had used Markov modelling to extrapolate available data to calculate lifetime costs and life expectancy. The reason for this is that the life expectancy of a patient on ART is a key determinant of lifetime costs and of the number of patients surviving and remaining in care over any projection period; it is therefore one of the most important inputs into any estimation of the costs of scaling up. A previous study by our group showed that 20% more patients would be remaining in care after a scale-up period of 10 years if life expectancy on ART of 13 years were assumed instead of 8.5 years [6]. However, we also included studies that attempted to ascertain the variables influencing costs over shorter time frames given that these could provide input into the construction of Markov states. A Pubmed search using the keywords “cost”, “resource-poor”, “low-income country/countries”, “middle-income country/countries”, “developing” and “antiretroviral” was conducted for all papers published before 1 May 2009.

### Model comparison and refinement

#### Data source

The determinants of costs and survival on ART were evaluated using a large database of patients enrolled with Aid for AIDS (AfA), a group that manages HIV-related care for a number of medical insurance funds in the private health care sector in Southern Africa. Aid for AIDS does not manage patients directly, but rather provides guidelines for private medical practitioners' care of its participants and reimburses claims. Medical care is provided via patients' own general practitioner, therefore there are no formal "sites" but rather several thousand general practitioners and specialist practices taking care of patients, including those participating in Aid for AIDS. Treatment is funded by contracted companies or medical aid funds (composed of pooled monthly contributions from members) which substantially cover co-morbid conditions including those not related to HIV. Data collected by AfA include demographics and previous medical history, CD4+ cell count, viral load and claims (including ART dispensing data). These claims are captured monthly by AfA from the medical insurance funds, pathology laboratories or from the patients or treating doctors directly using routine electronic administrative systems. Claim reimbursement is subject to established AfA protocols, including protocols for ART initiation, change of ART regimen, and the treatment of certain opportunistic infections. Despite this being a private sector programme, antiretroviral guidelines are similar in many respects to WHO guidelines for resource-poor settings as well as the South African public sector guidelines [2,7]. The recommended initial regimen is a combination of two nucleoside reverse transcriptase inhibitors (NRTIs) and a non-nucleoside reverse transcriptase inhibitor (NNRTI). Second line therapy consists of a boosted protease inhibitor (PI) with two NRTIs. Health services provided for patients within AfA include additional primary care doctor visits for patients that have exceeded their routine medical insurance benefits, telephonic counselling services and antiretroviral drugs dispensed monthly from private pharmacies or delivered via courier to the patient’s home. While ART can be initiated at CD4 <350 cells/µL rather than CD4 <200 cells/µL, we restricted the analysis to the latter group as this is the more common starting criterion in resource poor settings, including the South African public sector at the time of this study (these guidelines have recently been revised to recommend ART initiation at CD4 <350 cells/µL [8]). Furthermore, we only included patients starting ART with a NNRTI plus two NRTIs, as recommended by the WHO for resource-limited settings [2].

We determined average adherence to ART using monthly pharmacy refill data. This approach has been shown to correlate well with adherence assessment by therapeutic drug levels [9,10], and has been found to reliably predict virologic suppression [11], development of HIV drug resistance [12], and survival [13]. Previous analyses of these AfA data have in addition shown that this measure of adherence is a determinant of costs [14,15]. **We expressed pharmacy claim adherence as a percentage and calculated it as the number of months with ART claims submitted divided by the number of complete months from ART initiation to death, withdrawal from the Aid for AIDS program, or study end.**

Two of the medical insurance funds that contract AfA were selected on the grounds that they had large numbers of patients, similar treatment benefits, and no co-payment for ART. This allowed us to describe determinants of costs and outcomes without biases associated with the patient’s ability to pay, which has been reported to influence access to health care and outcomes on ART [16,17]. Patients were included in the study if they were ART naïve at entry (the exception being women who had received prophylaxis for prevention of mother-to-child transmission), adult (19 years or older at the time of approval for ART) and if ART was started between November 1998 and November 2007.

Direct health care costs were analysed from the provider’s perspective. The tariff amount was used as a proxy for these costs, as opposed to the amount charged by the provider. This is because providers may charge different rates for services with the same tariff code. The use of the tariff rate allows for the same cost to be assumed for the same type of service.

The prices of antiretroviral drugs have fallen dramatically over the past ten years. To account for this we deflated ARV prices to the April 2007 level. All other health care costs have increased; these were inflated to the April 2007 level using the Consumer Price Index net of mortgage payments (CPIX) [18]. The average South African Rand to United States Dollar (US$) exchange rate in April 2007 (R7.14 to US$1) was used to convert costs to US$ equivalents [19].

#### Establishment of Markov states

As the distribution of mean health care costs was right-skewed in our data, ordinary least squares regression was not appropriate [20,21]. Generalised linear regression models (GLM) have been proposed as they determine the impact of variables on the arithmetic mean and thus provide a method for identifying variables strongly associated with varied costs [20]. A GLM with a log-link function and a gamma distribution described the trends in the data well. To account for multiple measures within an individual as well as potentially strong correlations between the variables, we used generalised estimated equations with an unstructured correlation matrix. Variable coefficients and their 95% confidence intervals were determined using robust standard errors and the model fit was evaluated using deviance residuals [20].

Multiple Cox proportional hazard regression analysis was used to identify variables associated with a likelihood of dying. Based on the findings of both the cost and outcome analyses, a pragmatic decision on which Markov states to include in the final model is needed; as the number of states increases, so the model complexity increases exponentially. Data storage, basic calculations and data extraction was handled in Microsoft Sequel Server 2008. Statistical analysis was performed in Stata 10.

### Ethics statement

The study was approved by the Research Ethics Committee, University of Cape Town and by the Board of Directors of Aid for Aids. All patients signed consent for their information to be entered into the AfA database.

## Results

### Existing models in literature

Over 300 cost, cost-effectiveness and cost-utility analyses of ART were found via a Pubmed search, but these included only 6 different Markov models of ART in low and middle income countries, some of which were used in more than one publication [6,22-27]. A number of variables were used in these studies to define Markov states, as outlined in Table 1. These included the baseline (i.e. pre-ART) CD4 count category, the current CD4 count category, baseline and current viral load categories, time on ART, being on a first or second line ARV regimen, opportunistic infections or WHO staging; and adverse events on ART. These variables could be combined in a variety of ways to create distinct Markov states depending on the model.

**Table 1:** Determinants of Markov states in the literature

Markov states defined by:	Bachmann 2006	Badri et al 2006	Cleary et al 2006; 2008	Goldie et al 2006	Marseille et al 2006	Vijayaraghavan et al 2007
Baseline CD4 stratum		<200, 200-350, >350	<50, 50-200	<50; 50-200; 200-500; >500		
Current CD4 stratum	<200, 200-350	<200, 200-350, >350		<50; 50-200; 200-500; >500		
Baseline viral load stratum				>100,000; 30,001-100,000; 10,001-30,000; 3,001-10,000; 501-3,000; 0-500		
Current viral load stratum				>100,000; 30,001-100,000; 10,001-30,000; 3,001-10,000; 501-3,000; 0-500		Suppressed; unsuppressed
Time on ART			0-3; 3-6; 6-12; 12-24; 24-36; >36 months			
First or second line ARV regimen		Yes	Yes	Yes	Yes	Yes
Disease staging or opportunistic infections	Tuberculosis, other opportunistic infection, no opportunistic infection	No-AIDS/ AIDS		Severe bacterial infection; severe fungal infection; severe malaria; tuberculosis; isosporiasis; cerebral toxoplasmosis; nontuberculous mycobacteriosis; other severe opportunistic infection; mild bacterial infection; mild fungal infection; other mild infection	WHO Stages (1, 2, 3, 4)	AIDS
Adverse events						Toxicity; no toxicity

### Model comparison and refinement

#### Dataset

The characteristics of the cohort are described in Table 2. After exclusions, 5,177 patients met our eligibility criteria, with over 136,600 patient months of observation, about half of which were on ART. Median follow-up on ART was 19 months (IQR: 10 to 32). The proportion of patients who left the medical insurance fund was 34%. These patients either changed their employment, switched to a different medical insurance scheme or voluntarily stopped their contributions to the insurance scheme. The most common first line antiretroviral regimen was zidovudine/lamivudine/efavirenz (65 %). Lopinavir/ritonavir/zidovudine/didanosine was the most common second line regimen. CD4 and viral load monitoring was done 1.5 times per annum on average.

**Table 2:** The characteristics of the cohort (IQR = interquartile range)

Total number of patients		5 197
Patient months	Overall On ART	136 672 116 306 (85%)
Duration on ART (months)	Median IQR	19 (10 to 32)
Age at starting ART (years)	Median IQR	37.3 years (32.4 to 42.9)
Sex	Female Male	6 379(59.%) 4 356 (41%)
Patient status at end of study period	Active Left the scheme Dead	2 922 (56%) 1 834 (34%) 421 (8%)
Baseline CD4+ cell count	Median IQR	87 cells/μL (37 to 145)
Baseline viral load (log_10_)	Median IQR	5.22 (4.73 to 5.63)
NNRTI used in first line	Nevirapine Efavirenz	2 655 (26%) 6 711 (74%)
NRTI combination in first line	Zidovudine + lamivudine Stavudine + lamivudineother other	3 339 (65%) 1 225 (24%) 633 (11%)
Duration of CD4+ cell count monitoring before starting ART (months)	Median IQR	1.2 (0.6 to 2.6)
Overall Adherence as measured by monthly pharmacy refill data	Median IQR	74.4% (38.4 to 92.3)

IQR=Interquartile Range

#### Markov states

To determine the most important variables on which to base Markov states, we assessed whether variables had a sizeable effect on costs or on the likelihood of dying. The literature review identified a number of differences in the ways that variables were categorised. Using the categories described in the literature as a starting point, we determined the most appropriate categories for the variables in our dataset guided by residual diagnostics, whether overall model fit improved with a changed categorisation, and whether the p-value was significant at the 95% confidence interval. The variables included in the analysis were: (1) baseline CD4+ cell count (categorised as 0-49, 50-199 cells/µL) (following Cleary et al [6,22] and Goldie et al [25]); (2) current or most recent (carried forward for up to 12 months) CD4+ cell count (0-49, 50-199, 200 to 349, 350 to 499, and ≥500 cells/µl) (similar categories as Bachman [24], Badri et al [23] and Goldie et al [25]); (3) baseline viral load (categorised below or above 100,000 copies/ml) (Goldie et al [25] include this variable, but use far more categories); (4) Current or most recent viral load (categorised as <400, 400-10,000, 10,000-100,000, >100,000 copies/ml) (Goldie et al [25] and Vijayaraghavan et al [26] include this variable, but use different categories); (5) ART regimen (either first line or second line) (used by all except Bachman [24]); and (6) time periods relative to ART initiation (-4 to 4, 4 to 12, 12 to 24, 24 to 48 months on treatment) (similar to Cleary et al [6,22]). In addition to the variables identified in the literature, we also considered: (7) overall adherence, as determined using monthly pharmacy claim data and divided into quartiles based on the observed distribution of adherence in the cohort (<42%, 42%-75%, 75%-92%, >92%); (8) the NNRTI included in the initial first line regimen (either efavirenz or nevirapine); (9) the duration of CD4 count monitoring prior to starting ART, a proxy for duration within pre-ART care (≤6 months and > 6 months); (10) age at starting ART (<25, 25 to 50 and >50 years old); and (11) sex. Our data did not allow us to include variables relating to WHO disease staging, opportunistic infections or adverse events.

All the variables were included in the analysis exploring the determinants of costs and the likelihood of dying. In the baseline, the following initial parameter states were assumed: (1) baseline CD4 cell count between 50 and 199cells/µL; (2) current CD4+ cell count between 50 and 199cells/µL; (3) baseline viral load < 100,000 copies/ml; (4) current viral load < 400 copies/ml; (5) on first line ART; (6) time period 12 to 23 months on ART; (7) overall adherence in the upper quartile (>75%); (8) NNRTI = efavirenz; (9) >6 months of CD4 monitoring prior to starting ART; (10) age at starting ART 25 to 50; and (11) sex = female

**Figure 1:**
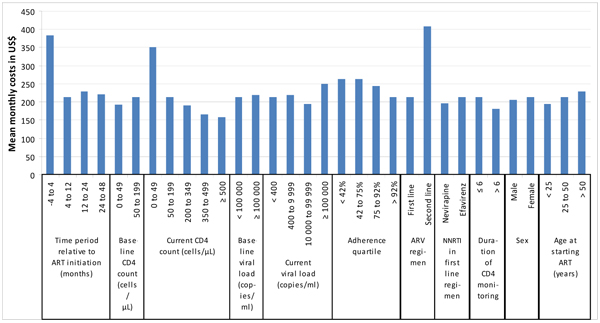
The influence of variables on mean monthly costs

The results from the multiple regression analysis of costs (all variables were included in the model) are found in Table 3. Mean cost per patient month is presented for each variable or variable category and is compared to a reference or baseline which is the mean cost across all patients and time periods. We found higher current CD4 counts were associated with lower costs while having a current viral load above 100,000 copies/ml was associated with increased costs. Being on efavirenz was more costly than nevirapine, and being on second line was more costly than being on first line; these findings are likely to relate to the higher costs of the ARV drugs in these states as opposed to other health care costs. Lower adherence was an important driver of costs, as was time on treatment, with monthly costs in the period from 4 months before starting ART to 4 months on ART being almost double the monthly costs thereafter. After this time, the size and significance of the association between costs and time on treatment waned dramatically. Figure 1 illustrates the full set of variables and their influence on the mean total monthly direct health care costs relative to the baseline scenario.

**Table 3:** Multiple generalised linear regression analysis of the determinants of total mean monthly costs (US$)

Variables		Value	% change from referent	p-value
Time period relative to ART initiation (months)	-4 to 4	384 (321 to 459)	80%	<0.001
	4 to 12	238 (204 to 277)	12%	0.158
	12 to 24	Referent		
	24 to 48	222 (182 to 270)	4%	0.705
Baseline CD4 count (cells/µL)	0 to 49	193 (164 to 227)	-10%	0.228
	50 to 199	Referent		
Current CD4 count (cells/µL)	0 to 49	352 (290 to 426)	65%	<0.001
	50 to 199	Referent		
	200 to 349	190 (161 to 223)	-11%	0.16
	350 to 499	166 (141 to 195)	-22%	0.002
	≥500	158 (123 to 202)	-26%	0.017
Baseline viral load (copies/ml)	<100 000	Referent		
	≥100 000	220 (195 to 248)	3%	0.601
Current viral load (copies/ml)	<400	Referent		
	400 to 9 999	219 (191 to 250)	3%	0.717
	10 000 to 99 999	195 (164 to 232)	-8%	0.317
	≥100 000	249 (209 to 296)	17%	0.082
Overall adherence	<42%	264 (235 to 297)	24%	<0.001
	42 to 74%	263 (233 to 295)	23%	0.001
	75 to 92%	244 (218 to 273)	14%	0.019
	≥92%	Referent		
ARV regimen	First line	Referent		
	Second line	409 (255 to 656)	92%	0.007
NNRTI in first line	Nevirapine	197 (182 to 213)	-8%	0.042
	Efavirenz	Referent		
Duration of CD4 count monitoring (months)	≤6	181 (161 to 204)	-15%	0.008
	>6	Referent		
Sex	Male	206 (188 to 226)	-3%	0.463
	Female	Referent		
Age at starting ART (years)	<25	194 (164 to 230)	-9%	0.282
	25 to 50	Referent		
	>50	230 (194 to 271)	8%	0.384
**Referent cost**		213 (178 to 256)	NA	NA

The results from the multiple Cox proportional hazards regression analysis of the likelihood of dying are found in table 4. The relative likelihood of dying is compared with the same referent groups as in the cost analysis, but a separate model was used for each of the time periods. We found higher current CD4 counts, higher ART adherence, and current viral loads below 100,000 copies/ml were associated with lower likelihoods of dying across all periods, with baseline values for CD4 and viral load contributing very little additional effect after the first 4 months on treatment. Longer duration of monitoring prior to starting ART was associated with a lower likelihood of dying in earlier periods and being greater than 50 years or younger than 25 years at starting ART was associated with a higher likelihood of dying in later periods. Being on second line was associated with an increased likelihood of dying across all periods.

**Table 4:** Multiple Cox proportional hazard regression analysis of the relative risk of dying

Variables		0 to 4 months on ART		5 to 12 months on ART		13 to 24 months on ART		>24 months on ART	
		
		co-eff (95% CI)	p-value	co-eff (95% CI)	p-value	co-eff (95% CI)	p-value	co-eff (95% CI)	p-value
Baseline CD4 cell count (cells/µL)	<50	2.09 (1.02 to 4.28)	0.043	1.6 (0.84 to 3.06)	0.152	1 (0.57 to 1.73)	0.992	0.79 (0.51 to 1.22)	0.279
	50-199	Referent							
Current CD4 cell count (cells/µL)	<50	N/A	N/A	1.89 (0.96 to 3.72)	0.065	3.88 (2.03 to 7.41)	<0.001	3.54 (2.17 to 5.75)	<0.001
	50-199	Referent							
	200-349	N/A	N/A	0.58 (0.25 to 1.34)	0.206	0.19 (0.06 to 0.55)	0.002	0.74 (0.38 to 1.44)	0.376
	≥350	N/A	N/A	N/A	N/A	0.36 (0.12 to 1.09)	0.071	0.42 (0.15 to 1.17)	0.098
	≥500	N/A	N/A	N/A	N/A	N/A	N/A	0.42 (0.15 to 1.23)	0.115
Baseline viral load (copies/ml)	<100 000	Referent							
	≥100 000	2.12 (0.86 to 5.21)	0.103	0.82 (0.44 to 1.53)	0.541	1.34 (0.77 to 2.33)	0.298	1.2 (0.78 to 1.85)	0.413
Current viral load (copies/ml)	<400	Referent							
	400 – 9 999	N/A	N/A	1.09 (0.44 to 2.66)	0.270	1.42 (0.57 to 3.59)	0.967	1.32 (0.56 to 3.14)	0.033
	10 000 – 99 999	N/A	N/A	0.64 (0.29 to 1.41)	0.106	1.02 (0.44 to 2.36)	0.394	2.24 (1.07 to 4.7)	0.001
	≥100 000	N/A	N/A	1.76 (0.89 to 3.47)	0.541	1.42 (0.63 to 3.2)	0.298	3.6 (1.68 to 7.7)	0.413
ART adherence (quartiles)	<42%	2.14 (0.69 to 6.67)	0.188	1.89 (1 to 3.57)	0.049	1.40 (0.59 to 3.29)	0.443	2.21 (0.87 to 5.63)	0.096
	42 to 75%	3.18 (1.05 to 9.61)	0.04	1.78 (0.92 to 3.43)	0.086	0.65 (0.26 to 1.64)	0.362	2.58 (1.04 to 6.39)	0.041
	75 to 92%	N/A	N/A	1.76 (0.9 to 3.46)	0.098	0.91 (0.37 to 2.23)	0.84	1.48 (0.56 to 3.91)	0.427
	>92%	Referent							
ARV regimen	First line	Referent							
	Second line	6.39 (0.85 to 47.75)	0.071	5.25 (2.11 to 13.06)	<0.001	1.4 (0.64 to 3.06)	0.402	1.67 (1.09 to 2.56)	0.018
NNRTI in first line	efavirenz	Referent							
	neviripine	0.6 (0.28 to 1.26)	0.175	1.26 (0.8 to 2)	0.324	1.56 (0.85 to 2.85)	0.149	0.89 (0.59 to 1.34)	0.568
Duration of monitoring prior to starting ART (months)	≥6	Referent							
	<6	2.9 (0.28 to 1.26)	0.296	1.15 (0.8 to 2)	0.692	1.67 (0.85 to 2.85)	0.328	1.05 (0.59 to 1.34)	0.890
Sex	male	0.79 (0.38 to 1.67)	0.545	1 (0.68 to 1.46)	0.998	1.15 (0.69 to 1.91)	0.595	1.26 (0.85 to 1.86)	0.257
	female	Referent							
Age at baseline (years)	<25	0.63 (0.15 to 2.7)	0.531	2.06 (0.51 to 8.4)	0.312	1.82 (0.25 to 13.22)	0.555	2.15 (0.52 to 8.83)	0.289
	25-49	Referent							
	≥50	1.69 (0.32 to 9.03)	0.540	2.39 (0.52 to 10.98)	0.263	1.34 (0.14 to 13.03)	0.801	3.86 (0.81 to 18.35)	0.090

## Discussion

We analysed determinants of direct health care costs and survival in 5,197 HIV-infected adults enrolled in a South African managed care ART programme with 136,672 patient months of follow-up, spanning -4 months before ART to 4 years on ART.  If each Markov state is defined to include unique combinations of these variables, thousands of states could be defined. A correspondingly large dataset would then be needed to calculate the costs and transitions associated with each of these states. For this reason, it becomes necessary to focus only on variables that have the most marked effects on costs and outcomes within reasonable confidence intervals. The key variables associated with changes in mean monthly costs were: being on the second line regimen; receiving ART from 4 months prior to 4 months post treatment initiation; having a recent or current CD4 count <50 cells/µL or 50-199 cells/µl; having mean ART adherence <75% as determined by monthly pharmacy refill data; and having a current or recent viral load >100,000 copies/mL. In terms of the likelihood of dying, the key variables associated with changes in survival were: baseline CD4 count<50 cells/µl (particularly during the first 4 months on treatment); current CD4 count <50 cells/µl and 50-199 cells/µl (particularly during later periods on treatment); and being on the second line regimen. Being poorly adherent and having an unsuppressed viral load was also associated with a higher likelihood of dying.

The relationships between these variables and costs and outcomes were consistent with trends described in the literature, though the scale did differ in some cases: lower CD4 count, higher viral load, lower adherence, and being on second line therapy was associated with higher costs and worse outcomes. In addition, sub-optimal adherence drives resistance to first line therapy, leading to second line therapy being initiated. There was a relative small and limited association between costs and outcomes and the baseline pathology results (CD4 count and the viral load), most likely due to the current or most recent CD4 and viral load results being dominant. The finding that lower adherence is associated with higher costs (in addition to its known effects on biological variables such as CD4 count and viral load and starting second line therapy) further supports the need to include this variable in Markov models.

Based on the above, one would anticipate that Markov models that use these variables as the bases of their Markov states may have superior accuracy. However, in our literature review, the only Markov model that specified costs and outcomes in relation to duration on ART was Cleary et al [6,22]. Most of the models separated first from second line ART to capture the cost differences, but were unable to estimate different survival transition probabilities because data on outcomes of patients on second line was limited. Bachmann [24], Goldie et al [25] and Badri et al [23] all included the current CD4 count but Cleary et al [6,22] only included the baseline CD4 count. Baseline and current viral load was only included by Goldie et al [25]. Opportunistic infections or WHO staging were included in all the models except Cleary et al [6,22]. Unfortunately these data were not available within the AfA cohort, and we were therefore unable to assess the importance of these for the construction of Markov states. None of the models included sex, ART adherence, age or duration of pre-ART care.

There are a number of limitations to this analysis. First, our cohort consisted of private sector patients when the majority of patients in resource-limited settings are treated in the public or NGO sector. However, the baseline characteristics of our cohort (CD4+ cell count, proportion female and age) are comparable with cohorts from low-income countries [28] and we restricted our analysis to patients receiving NNRTI-based first line ART regimens and starting ART at CD4<200 cells/µl, in keeping with WHO recommendations for resource-limited settings [2]. While we would not claim that our actual cost findings are generalisable to public sector settings or to other countries, we would argue that the variables that influence costs and outcomes are likely to be relevant even if the magnitude of their effects could differ. Second, the impact of specific opportunistic infections, disease staging or adverse events on costs and outcomes was not included in this analysis as these data were not available. We are therefore unable to comment on the validity of this aspect of some of the Markov models in the literature.

## Conclusion

In conclusion, we have analysed the determinants of direct health care costs and outcomes in a private health care sector managed ART programme. Our focus has been to use statistical techniques to determine the key variables to include in Markov states and to use these findings to inform future modelling of the costs of scaling up ART. Our results suggest that important drivers of costs and outcomes include time on ART, being on first versus second line regimens, the current CD4 cell count, the current viral load, age at starting ART and adherence. The inclusion of these variables should be considered for future modelling of the costs of scaling up ART programmes.

## Competing interests

The authors declare that they have no competing interests.

## Authors’ contributions

RL analyzed the data. MH collected and prepared the data used in the study and provided input into the data analysis methodology. SC, GM and RL contributed to the overall study design and methodology. SC wrote the first draft of the paper while SC and RL were responsible for revisions of the paper. All authors contributed to the writing of the paper and agreed with the manuscript’s results and conclusions.

## References

[B1] Cleary SM McIntyre D Affordability - the forgotten criterion in health care priority setting Health Economics 2009 18 4 373 375 10.1002/hec.1450 19267322

[B2] WHO Antiretroviral therapy for HIV infection in adults and adolescents in resource-limited settings: towards universal access: guidelines for a public health approach. Geneva: World Health Organization 2006

[B3] Sonnenberg FA Beck JR Markov models in medical decision making: a practical guide Medical Decision Making 1993 13 4 322 338 10.1177/0272989X9301300409 8246705

[B4] Briggs A Sculpher M An Introduction to Markov Modelling for Economic Evaluation Pharmacoeconomics 1998 13 4 397 409 10.2165/00019053-199813040-00003 10178664

[B5] Cleary S Mooney G McIntyre D Equity and efficiency in HIV-treatment in South Africa: the contribution of mathematical programming to priority setting Health Econ 2009 DOI: 10.1002/hec.1542 10.1002/hec.1542 19725025

[B6] Cleary SM McIntyre D Boulle AM Assessing efficiency and the costs of scaling-up HIV-treatment AIDS 2008 22 Suppl 1 S35 S42 10.1097/01.aids.0000327621.24232.71 18664951

[B7] National Antiretroviral Treatment Guidelines. Pretoria: National Department of Health 2004

[B8] Thom A Health Department approves new HIV treatment guidelines Health-e 2010

[B9] Steiner J Prochazka A The assessment of refill compliance using pharmacy records: methods, validity, and applications Journal of Clinical Epidemiology 1997 50 105 116 10.1016/S0895-4356(96)00268-5 9048695

[B10] Steiner J Koepsell T Fihn S Inui T A general method of compliance assessment using centralized pharmacy records: description and validation Med Care 1988 26 814 823 10.1097/00005650-198808000-00007 3398608

[B11] Low-Beer S Yip B O'Shaughnessy M Hogg R Montaner J Adherence to triple therapy and viral load response 2000 JAIDS 2000 23 360 361 10836763 10.1097/00126334-200004010-00016

[B12] Harrigan P Hogg R Dong W Yip B Wynhoven B Woodward J Predictors of HIV drug-resistance mutations in a large antiretroviral-naive cohort initiating triple antiretroviral therapy Journal of Infectious Diseases 2005 191 339 347 10.1086/427192 15633092

[B13] Nachega J Hislop M Dowdy D Lo M Omer S Regensberg L Chaisson R Maartens G Adherence to highly active antiretroviral therapy assessed by pharmacy claims predicts survival in HIV-infected South African adults JAIDS 2006 43 78 84 16878045 10.1097/01.qai.0000225015.43266.46

[B14] Nachega JB Leisegang R Bishai D Nguyen H Hislop M Cleary S Regensberg L Maartens G Association of Antiretroviral Therapy Adherence and Health Care Costs Annals of Internal Medicine 2010 152 Mar 18 25 20048268 10.7326/0003-4819-152-1-201001050-00006

[B15] Leisegang R Cleary S Hislop M Davidse A Regensberg L Little F Maartens G Early and Late Direct Costs in a Southern African Antiretroviral Treatment Programme: A Retrospective Cohort Analysis PloS Medicine 2009 6 12 e1000189. doi:1000110.1001371/journal.pmed.1000189. 10.1371/journal.pmed.1000189 19956658 PMC2777319

[B16] Ivers L Kendrick D Doucette K Efficacy of antiretroviral therapy programs in resource-poor settings: a meta-analysis of the published literature Clinical Infectious Diseases 2005 41 2 217 224 10.1086/431199 15983918

[B17] Brinkhof M Dabis F Myer L Early loss of HIV-infected patients on potent antiretroviral therapy programmes in lower-income countries Bulletin of the World Health Organization 2008 86 7 559 567 10.2471/BLT.07.044248 18670668 PMC2647487

[B18] Statistics South Africa Historical CPIX key indicators. STATSSA 2009

[B19] Federal Reserve Statistical Release: Foreign Exchange Rates (Annual) http://www.federalreserve.gov/releases/g5a/

[B20] Thompson S Barber J How should cost data in pragmatic randomised trials be analysed? British Medical Journal 2000 320 7243 1197 1200 10.1136/bmj.320.7243.1197 10784550 PMC1127588

[B21] Dodd S Bassi A Bodger K Williamson P A comparison of multivariable regression models to analyse cost data Journal of Evaluation in Clinical Practice 2006 12 1 76 86 10.1111/j.1365-2753.2006.00610.x 16422782

[B22] Cleary SM McIntyre D Boulle AM The cost-effectiveness of Antiretroviral Treatment in Khayelitsha, South Africa: a primary data analysis Cost Eff Resourc Alloc 2006 4 20 10.1186/1478-7547-4-20 PMC1770938 17147833

[B23] Badri M Cleary S Maartens G Pitt J Bekker L-G Orrell C Wood R When to initiate HAART in sub-Saharan Africa? A South African cost-effectiveness study Antiviral Therapy 2006 11 63 72 16518961

[B24] Bachman M Effectiveness and cost effectiveness of early and late prevention on HIV/AIDS progression with antiretrovirals or antibiotics in Southern African adults AIDS Care 2006 18 2 109 120 10.1080/09540120500159334 16338768

[B25] Goldie S Yazdanpanah Y Losina E Weinstein M Anglaret X Walensky R Hsu H Kimmel A Holmes C Kaplan J Cost-Effectiveness of HIV Treatment in Resource-Poor Settings - The Case of Cote d'Ivoire New England Journal of Medicine 2006 355 11 1141 1153 10.1056/NEJMsa060247 16971720

[B26] Vijayaraghavan A Efrusy M Mazonson P Ebrahim O Sanne I Santas C Cost-Effectiveness of Alternative Strategies for Initiating and Monitoring Highly Active Antiretroviral Therapy in the Developing World JAIDS 2007 46 1 91 100 17621241 10.1097/QAI.0b013e3181342564

[B27] Marseille E Saba J Muyingo S Khan JG The costs and benefits of private sector provision of treatment to HIV-infected employees in Kampala, Uganda AIDS 20 907 914 10.1097/01.aids.0000218556.36661.47 16549976

[B28] ART-LINC ART-CC Mortality of HIV-1-infected patients in the first year of antiretroviral therapy: comparison between low-income and high-income countries Lancet 2006 367 817 824 10.1016/S0140-6736(06)68337-2 16530575

